# High prevalence of co-infection between human papillomavirus (HPV) 51 and 52 in Mexican population

**DOI:** 10.1186/s12885-017-3519-7

**Published:** 2017-08-08

**Authors:** Jazbet Gallegos-Bolaños, Jessica Alejandra Rivera-Domínguez, José Miguel Presno-Bernal, Rodolfo Daniel Cervantes-Villagrana

**Affiliations:** 1 Departamento de Genómica y Biología Molecular, Laboratorio Carpermor, 06470 CDMX, Mexico; 2 Dirección de Proyectos e Investigación, Grupo Diagnóstico Médico Proa, 06400 CDMX, Mexico; 3 Departamento de Investigación Clínica, Grupo Diagnóstico Médico Proa, 06400 CDMX, Mexico; 4 Departamento de Farmacología, Centro de Investigación y de Estudios Avanzados del IPN (CINVESTAV-IPN), 07360 CDMX, Mexico

**Keywords:** Co-infection, Human papillomavirus, HPV, HPV 51, HPV 52, Genotypes, Prevalence, Mexico

## Abstract

**Background:**

Human papillomavirus (HPV) is associated with the genesis of cervical carcinoma. The co-infection among HPV genotypes is frequent, but the clinical significance is controversial; in Mexico, the prevalence and pattern of co-infection differ depending on the geographic area of study. We analyzed the mono- and co-infection prevalence of multiple HPV genotypes, as well as preferential interactions among them in a Mexico City sample population.

**Methods:**

This study was designed as a retrospective cohort study. Cervical cytology samples from 1163 women and 166 urethral scraping samples of men were analyzed between 2010 and 2012. The detection of HPV infection was performed using the hybrid capture and the genotyping was by PCR (HPV 6, 11, 16, 18, 30, 31, 33, 35, 45, 51, and 52).

**Results:**

36% of women were HPV-positive and the most prevalent genotypes were HPV 51, 52, 16, and 33 (42, 38, 37, and 34%, respectively). The prevalence of co-infection was higher (75.37%) than mono-infection in women HPV positives. All genotypes were co-infected with HPV 16, but the co-infection with 51–52 genotypes was the most frequent combination in all cases.

**Conclusion:**

The co-infection was very common; each HPV genotype showed different preferences for co-infection with other genotypes, HPV 51–52 co-infection was the most frequent. The HPV 16, 33, 51 and 52 were the most prevalent and are a public health concern to the Mexican population.

**Electronic supplementary material:**

The online version of this article (doi:10.1186/s12885-017-3519-7) contains supplementary material, which is available to authorized users.

## Background

The human papillomavirus (HPV), belonging to the *Papillomaviridae* family, is an infectious agent of epithelial tissue with high clinical relevance for its association with the generation of cervical carcinoma [1–3]. There are over 150 different HPV genotypes described in humans, which are viruses with a double-stranded circular DNA containing 8000 base pairs associated to histones [2, 4, 5]. The HPV-DNA integration into the infected cell genome is a key event for malignant transformation of host cells [6, 7]. The International Agency for Research on Cancer (IARC) states that visible genital warts are caused by low-risk HPV (LR-HPV) genotypes such as 6, 10, 11, 32, 42, 43, 44, and 61, which are not associated with cervical cancer [8, 9]. In contrast, HPV genotypes 16, 18, 30, 31, 33, 35, 39, 45, 51, 52, 56, 58, 59, 66, 67, and 68 are considered as high-risk (HR-HPV) and found in 98% of women with high-grade squamous intraepithelial lesion [4, 9, 10].

Co-infection among HPV types is common in women, [11] and men, [12, 13] but their clinical significance remains controversial and the epidemiology of HPV genotype combinations is unknown. Some studies show that co-infection increases cervical cancer risk; [3, 14] and the presence of multiple HPV types is associated with a low response and survival rate of patients with cervical cancer that are receiving radiotherapy [15]. However, other authors found no evidence of synergy for high-grade squamous intraepithelial lesions, [16] or observed a viral antagonism during co-infection, [17] which suggests that the interaction between HPV 16 and 18 shows a competitive integration into genomic DNA of host cells when co-infected [18].

It is important to determine the epidemiology of mono- and co-infections of HPV in order to establish appropriate prevention strategies for the design of new vaccines according to each population [19, 20]. In some countries, HPV co-infection is less frequent than the mono-infection, [21, 22] but others have a higher co-infection prevalence [13]. In Mexico the prevalence and patterns of co-infection differ according to the geographic location analyzed [23, 24]. However, the hypothesis that the HPV genotype prefers to co-infect with specific genotypes has not been evaluated. The aim of this study was to analyze the co-infection prevalence of multiple HPV genotypes and to identify the most frequent interactions among them within the Mexican population.

## Methods

### Specimen collection

This study was designed as a retrospective cohort study. Samples were obtained from patients that performed clinical tests for the diagnosis of HPV infection in Mexico City; endocervical cytology from women and urethral scrapings from men between 2010 and 2012 were analyzed. These data were not collected or used for another study and have not been previously published. The protocol of analysis of the data obtained for diagnostic purposes was reviewed and approved by Carpermor’s Laboratory Ethics Committee, and was conducted according to the ethical guidelines of the Declaration of Helsinki and to the Official Mexican Standard NOM-012-SSA3–2012, this study was risk free and all information of individuals was anonymized. The study included 1329 patients, of which 1163 were women aged 16 to 72 (31.65 ± 0.43), and 166 were men aged 21 to 68 years (36.07 ± 1.5). An endocervical brush and swab for urethral scraping were used, and the samples were placed in tubes with a Digene transport medium. Finally, they were frozen at −20 °C prior to analysis. All samples were obtained and processed properly; therefore, all data were included for analysis.

### Hybrid capture assay

To identify the HPV-positive patients, we used the Hybrid Capture II test (Digene) according to the manufacturer’s instructions. Hybridization was performed in a microplate with the samples and corresponding probes (probes LR-HPV were RNA of HPV 6, 11, 42, 43, and 44; and probes HR-HPV were RNA of HPV 16, 18, 31, 33, 35, 39, 45, 51, 52, 56, 58, 59, and 68). The hybridized samples were transferred to wells of capture microplate coated with anti-hybrid antibody (anti-RNA/DNA). Then, anti-RNA/DNA-alkaline phosphatase-conjugated and substrate dioxetane were added for detection in the luminometer. The HPV-positives samples (hybrid capture assay) were used to determine the viral genotype by PCR.

### HPV genotyping by PCR

DNA extraction was performed in 300–500 μL of each sample using QIAamp UltraSens Virus kit (Qiagen) according to the manufacturer’s instructions. On DNA viral amplification by PCR, we used specific primers for E6 and E7 region of HPV 6, 11, 16, 18, 30, 31, 33, 35, 45, 51, and 52 types (Invitrogen). For the mixture preparation, the multiplex PCR amplification kit (TaqMan) was used according to the manufacturer’s instructions. Then, the samples were placed in the thermocycler (GeneAmp PCR system 9700) and amplified during 45 cycles to the temperature corresponding to each primer. The amplified samples were loaded into 2% agarose gel and set in an electrophoresis chamber (Horizon 11–14, Gibco BRL) at 80 V for 45 min. Finally, the gel bands were observed with ethidium bromide in the transilluminator (MacroVue UVis-20, Hoefer) [25, 26].

### Statistical analysis

Proportions were calculated from the total number of analyzed patients: HPV-positive patients for any type, and HPV-positive patients for specific types. The analysis of proportions was performed using the z-test from the statistical software, Sigma Plot 11.0, and the graphs were made in GraphPad Prism 5 software. Differences were considered statistically significant for values of *p* < 0.05.

## Results

**Fig. 1:**
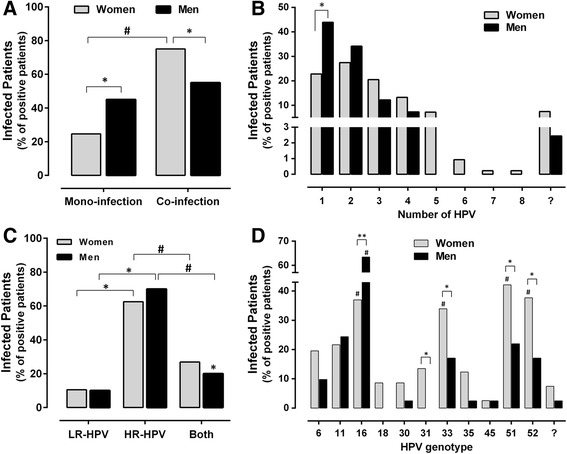
The co-infection prevalence was higher than the mono-infection in HPV-positive patients. **a** The HPV-positive patients with mono- or co-infection in women and men. #*p* < 0.001 (mono- vs co-infection), *p = 0.009 (women vs men), z-test. **b** Frequency of patients with single or multiple HPV genotypes. Number of genotypes involved in co-infection was of two to eight different genotypes detected. *p = 0.005 (women vs men), z-test. **c** The infected patient prevalence with LR-HPV, HR-HPV or both. *p < 0.001 (LR-HPV vs HR-HPV/both); #*p* < 0.001 (HR-HPV vs both), z-test. **d** The prevalence of HPV 6, 11, 16, 18, 30, 31, 33, 35, 45, 51, 52, and other HPV genotypes was not sensitive to the method of genotyping (?). *p < 0.05, **p < 0.01 (women vs men); #*p* < 0.001 (prevalence within each gender), z-test

The results were analyzed from 1329 patients samples (both genders): 858 (64.56%) were negative and 471 (35.44%) patients were HPV-positive in the hybrid capture test. From 1163 women evaluated, 36% were HPV-positive; while from the 166 men tested, 24% were positive (Additional file 1). We determined the percentage of patients with mono- and co-infection in each gender (Fig. 1a). Women had a higher prevalence of co-infection (75.37% of positive samples, ^#^ *p* < 0.001). We identified patients with co-infections of two or more HPV genotypes (Fig. 1b). Frequently, women had an infection with 1 to 5 different HPV genotypes, as well as with 6 HPV genotypes (four cases), 7 and 8 HPV genotypes (one case), yet this represents only the sensitive viruses for the genotyping test. Meanwhile, men had been infected with 1 to 4 different HPV genotypes. In some HPV-positive patients, the specific genotype was not identified by PCR, but the infection was detected by hybrid capture (LR-HPV 42 to 44, or HR-HPV 39, 56, 58, 59, and 68), and the prevalence of these genotypes was 7% in women and one case in men. The proportions among infections with LR-HPV, HR-HPV or both were statistically different in each gender (Fig. 1c). The number of patients with HR-HPV was higher than that of the patients with LR-HPV or both (LR- and HR-HPV).

The different HPV genotypes assessed had distinct prevalence (Fig. 1d); women had a higher prevalence of HPV 16, 33, 51, and 52 with similar frequency (37, 34, 42, and 38%, respectively) and were significantly different to other genotypes (#*p* < 0.001); in contrast, HPV 45 was less frequent (2.56%). In men, genotype 16 was found in 63.41% of patients (#*p* < 0.001), while others such as genotype 11 and 51 were found in ~22% of patients. HPV 18 and 31 were not identified in any of the men samples evaluated. When comparing genders, the infection frequency by genotypes 31, 33, 51, and 52 was higher in women than men (*p < 0.05); but the prevalence of HPV 16 was higher in men than women (**p < 0.01).

All evaluated HPV genotypes showed a preference for co-infection in women (Table 1). Over 80% of HPV-infected women presented a co-infection (*p < 0.001, Table 1: C-I column in women). In all samples with HPV 18 or 35, these genotypes were only present in co-infection with other genotypes (Table 1, C-I column for women). For men samples, HPV 11, 16, and 51 genotypes were found significantly in co-infection (^‡^ *p* < 0.05); however, the positive samples of men were too scarce for a conclusive statistical analysis of other genotypes.

**Table 1:** Prevalence of each HPV genotype identified in mono- and co-infection of women and men. First column correspond to HPV genotypes tested. Second column cluster correspond to women data of mono-infection, co-infection, and number samples of each genotype. Third column cluster correspond to men data of mono-infection, co-infection, and the number samples. Data represent as percentage of mono- or co-infection for each genotype

Type	Women			Men		
	M-I (%)	C-I (%)	n	M-I (%)	C-I (%)	n
6	17.86	82.14*	84	25	75	4
11	19.35	80.65*	93	20	80^‡^	10
16	13.21	86.79*	159	30.77	69.23^‡^	26
18	0	100*	37	0	0	0
30	18.92	81.08*	37	100	0	1
31	6.9	93.1*	58	0	0	0
33	6.16	93.84*	146	28.57	71.43	7
35	0	100*	53	0	100	1
45	18.18	81.82*	11	0	100	1
51	6.08	93.92*	181	11.11	88.89^†^	9
52	6.79	93.21*	162	42.86	57.14	4

M-I: Mono-Infection

C-I: Co-Infection

z-test: *p < 0.001, ^†^ *p* < 0.01, ^‡^ *p* < 0.05

**Fig. 2:**
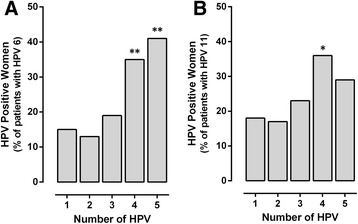
The frequency of low-risk HPV genotypes increased with the number of HPV genotypes in co-infection. Data represents the percentage of infected women with **a**) HPV 6 and **b**) HPV 11 in co-infection of 2, 3, 4, and 5 different genotypes *p < 0.05, **p < 0.01 (single- vs co-infection), z-test

The LR-HPV prevalence (genotypes 6 and 11) increased with the number of genotypes that co-infected in women. HPV 6 frequency increased with the number of genotypes that interacts (Fig. 2a), so that co-infections of 4 and 5 viral genotypes, was present in almost 40% of the subjects (**p < 0.01). The HPV 11 showed preference for co-infection with four genotypes (35%, *p < 0.05) (Fig. 2b).

**Fig. 3:**
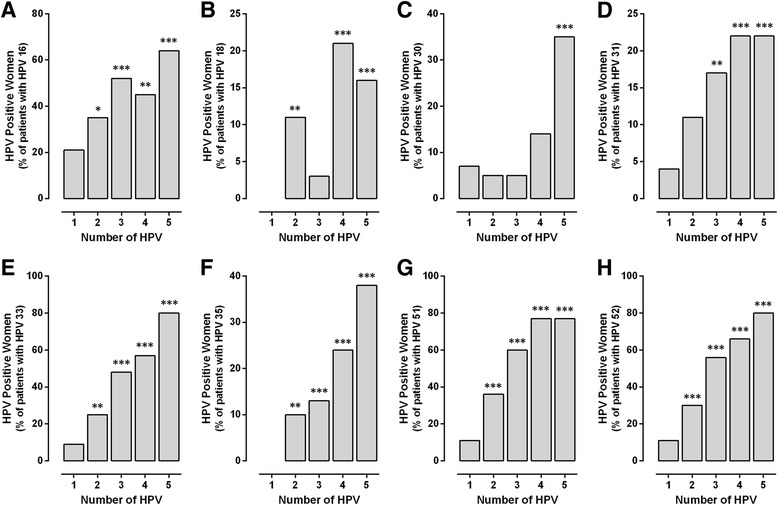
The frequency of high-risk HPV genotypes increased with the number of HPV genotypes in co-infection. Data represents the percentage of infected women with **a**) HPV 16, **b**) 18, **c**) 30, **d**) 31, **e**) 33, **f**) 35, **g**) 51, and **h**) 52 in co-infection of 2, 3, 4, and 5 genotypes *p < 0.05, *p < 0.01, ***p < 0.001 (single- vs co-infection), z-test

Differences were observed in the frequency of high-risk genotypes analyzed (HPV 16, 18, 30, 31, 33, 35, 51, and 52) in co-infection with others genotypes (Fig. 3). The HPV 16 prevalence increased significantly with the HPV number involved, mainly in co-infections of 3 and 5 viral genotypes (***p < 0.001), with maximum 64.5% of individuals infected by five HPV genotypes (Fig. 3a). There were no mono-infected patients with HPV 18, all positive samples for HPV 18 had co-infection with two or more different genotypes, and were significant to 2, 4, and 5 viral genotypes (Fig. 3b). Similarly, genotype 35 was found exclusively in co-infection, never as single infection, in co-infections of five genotypes, the HPV 35 had a frequency of 38.7% of women (Fig. 3f).

The HPV 30 and 31 were mainly found in co-infections of 5 viral genotypes and 4–5 genotypes, respectively (Fig. 3c and d). Interestingly, HPV 33 had a clear association with other genotypes and was detected in 80% of infected patients with five different genotypes, similar to HPV 51 and 52 types (77 and 80%, respectively). These three genotypes (HPV 33, 51, and 52) were postulated as genotypes with greater frequency and association (Fig. 3e, g, and h). Finally, HPV 45 did not show preference for some co-infections, perhaps due to their low prevalence and it is necessary to increase the number of positive samples to verify this data.

We analyzed if each genotype was preferably associated with another genotype; within the frequency of each genotype, it was possible to determine the preference for co-infection with another genotype (Additional file 2 and Table 2). All genotypes significantly co-infected with HPV 16, but did not have the higher frequencies (Table 2, HPV 16 column). The low-risk genotypes such as HPV 6 and 11 co-infected with HPV 16, 33, 51, and 52 frequently (*p* < 0.001). For the cervical samples with HPV 16 (the genotype strongly associated with cervical cancer), we found that the ~20% positive samples to HPV 16 co-infected mainly with HPV 33, 51 or 52 (*p* < 0.001, Table 2: HPV 16 row). The HPV 18 co-infects frequently with genotypes 51 and 52 (~30%, *p* < 0.001, Table 2: HPV 18 row), followed by the co-infection with HPV 33, 16, and 6. The genotypes 30 and 31 co-infected frequently with HPV 52, while HPV 33 co-infected significantly with HPV 6, 16, and 51, but not with genotype 52. Interestingly, HPV 35 co-infected specifically with HPV 16 and 6, but not with the most prevalent genotypes, HPV 51 and 52. Similar to above, the HPV 45 only co-infected significantly with HPV 16. The co-infection between HPV 51 and 52 was the most frequent combination (51.93 and 58.02%, respectively, Table 2: HPV 51 and 52 rows). Additionally, HPV 51 and 52 significantly co-infect with HPV 16 and 6.

**Table 2:** Interaction among HPV genotypes in infected women with two or more genotypes. First column correspond to HPV genotypes tested in women, each row show that percentage of frequency of co-infection with other genotypes identified in the next eleven columns. Last column show the number samples with each genotype. Data represent as percentage of co-infection for each genotype. *See* Additional file 2

Type	***6***	***11***	***16***	***18***	***30***	***31***	***33***	***35***	***45***	***51***	***52***	***n***
*6*	-	33.33*	28.57*	9.52	9.52	8.33	35.71*	15.48	1.19	39.29*	38.10*	84
*11*	30.11*	-	30.11*	4.3	4.3	5.38	17.20^†^	5.38	0	32.26*	20.43^†^	93
*16*	15.09*	17.61*	-	5.03	4.40	7.55	25.16*	13.84^†^	3.14	26.42*	20.13*	159
*18*	21.62^†^	10.81	21.62^‡^	-	0	13.51	24.32^‡^	0	5.41	27.03^†^	32.43^†^	37
*30*	21.62^†^	10.81	18.92^‡^	0	-	5.41	16.22^‡^	8.11	0	16.22^‡^	24.32^†^	37
*31*	12.07	8.62	20.69^†^	8.62	3.45	-	13.79	5.17	1.72	22.41^†^	27.59^‡^	58
*33*	22.05*	10.96	27.4*	6.16	4.11	5.48	-	3.42	0	15.07^†^	11.64	146
*35*	24.53^†^	9.43	41.51*	0	5.66	5.66	9.43	-	0	5.66	3.77	53
*45*	9.09	0	45.45^‡^	18.18	0	9.09	0	0	-	9.09	9.09	11
*51*	18.23*	16.57*	23.2*	5.52	3.31	7.18	12.15	1.66	0.55	-	51.93*	181
*52*	19.75*	11.73	19.75*	7.41	5.56	9.88	10.49	1.23	0.62	58.02*	-	162

z-test: ^‡^p < 0.05, ^†^p < 0.01, *p < 0.001

## Discussion

The HPV-infections are clearly associated with the development of cervical cancer and it is necessary to establish vaccine strategies to reduce the incidence of infections of all prevalent genotypes in each country. Our findings suggest that HPV 16, 33, 51, and 52 genotypes are of public health concern due to their high prevalence, similar to HPV prevalence found in Brazil [20] and in the Kingdom of Bahrain [19], and this is probably the cause of the increased incidence of cervical cancer in Mexico; recently, HPV 33 and 52 are considered in new vaccines [27]. We identified that 24% of the tested men were infected with HPV, a lower prevalence than women; while other authors found a higher prevalence (61.9%) of infected Mexican men, similar to the USA and less than Brazil [23]. The women showed a higher prevalence (36%) of infection than men, similar to results obtained in Italy where a prevalence of 35.9% HPV infected women was identified [28]. In Puebla, Mexico, a lower prevalence of infected women (25.4%) was identified, and some of the most common genotypes differ from our study: the HPV 16 (54.2%) had a higher prevalence than HPV 18 (37.3%), while HPV 31, 6, and 11 genotypes were less frequent (9.6, 9.6, and 4.8%, respectively) [11].

In southeastern Mexico, the low-risk genotypes (HPV 6 and 11) had a higher prevalence, [29] but in patients with cervical intraepithelial neoplasia, the HPV 16 and 58 were prevalent (30.6 and 24%, respectively); HPV 18 was not identified [30]. Meanwhile, in African countries, the prevalence of HPV 16, 33, and 58 was greater than that of other types [31]. In Mexican patients with cervical cancer, it was found that HPV 16 had a frequency of 71.6% and HPV 18 had only 4.6%; HPV 58 was found in 18.6% of patients with a high-risk squamous intraepithelial lesion [32]. In the colposcopies of patients with intraepithelial lesions and cervical cancer, HPV 58 had a high prevalence (28.5%), while 25.7% presented HPV 16 [33].

The above evidence establishes the correlation of HPV 16 and 58 in the carcinogenesis of Mexican women; meanwhile, HPV 18 appears to have no role [33]. Our results suggest that besides HPV 16 and 58, HPV 51 and 52 types may have a role in cervical carcinogenesis due to their high frequency, but further studies are necessary to support this data. A similar presence of HPV genotypes were found in Mexican soldiers with a high frequency of HR-HPV 52, 51, 16, and 58 types, and of LR-HPV 6, 11, 53, and 84 [34]. The variability of the viral prevalence among studies could be due to the geographical area of the selected population and the anatomical site of sampling. Particularly, a study found that in men, the HPV detection is better if the sample is from the external genital skin. Conversely, if the sample is obtained from the urethra and urinary meatus, the HPV detection potentially decreases [34]. These factors should be considered in future research and clinical practice.

The HPV co-infection with multiple genotypes probably promotes the progression of intraepithelial lesions and cervical cancer in Mexican women. We found that the co-infection among HPV genotypes was more frequent than mono-infection in the tested population, involving a greater number of viral genotypes (7 and 8 different genotypes identified in one case). In contrast, in another study from southeast Mexico, only 23.5% of HPV-positive patients showed multiple infections with 2 and 3 different genotypes [29]. In Italy, the co-infection is less frequent than the mono-infection, [28] as well as in Spain [35].

The genotypes 16 and 18 are classified as oncogenic in humans (group 2A), [9] our data showed a higher prevalence of genotype 16, with capacity to co-infect with all tested genotypes and probably a synergistic interaction in the carcinogenesis. In turn, the prevalence of HPV 18 was very low and all infected individuals with this genotype had co-infection. These results suggest that HPV 16 is the major genotype involved in cervical carcinoma in Mexican women. In the tested population, the genotypes 51 and 52 presented the higher prevalence, even exceeding HPV 16. Thus, in Mexico, the creation of a vaccine that provides protection against HR-HPV 51 and 52 is necessary. Similarly, a high frequency of co-infection of HPV 16 and 51 with different LR- and HR-HPV was found in Italy [28] and Brazil [20].

Currently, the clinical relevance of co-infection in the generation and progress of cervical cancer is unclear. It is likely to happen that initial infection with a particular genotype creates the best conditions for another genotype to infect, and without the first, a second one does not infect per se. This phenomenon may occur for HPV 18 and 35, genotypes only presented in co-infection (Table 2). In Colombian women, it was found that infection with HPV 16 or 18 increases the risk of getting an HPV 58 infection [21]. However, another study concluded that the risk factor for viral acquisition did not differ between mono- and co-infection, and stated that new infections were random [36].

In co-infections detected in the primary tumor, it was found that a viral genotype DNA integrates into the host genome, while the other was maintained in episomal form; [18] but in metastatic cells, co-infection remains and both genotypes were integrated into the genomic DNA [37]. The relevance of this remains unclear, but it is likely that integration of two or more viruses is essential for metastasis to start. Furthermore, it was determined that although the viral DNA remains at episomal state, it can generate chromosomal instability in the host cell [3]. In contrast, antagonism has been proposed by viral interference between a high- and low-risk HPV [17]. This suggests that in patients co-infected with high and low-risk HPV, it will be less likely to develop carcinoma.

The interaction among multiple HPVs may have implications in oncogenic risk [38]. In Brazil, researchers found that co-infection promotes cervical carcinogenesis; [14] and in Sweden the co-infection of HPV 16 and 18 is related to a higher risk of cervical adenocarcinoma in situ*,* and invasive generation [3]. A study in Italy showed that in patients with cervical intraepithelial neoplasia the most common co-infections were HPV 16–18 and 51–52, and also co-infections of three genotypes, such as HPV 16–51-52 [38]. In Mexico City, the co-infection of HPV 16 and 68 increases the risk of high-grade lesions and cervical cancer [39]. Our data showed that HPV 16, a genotype with high clinical relevance, co-infects with all genotypes tested, but the most common co-infection was HPV 51–52 (Table 3).

**Table 3:** Prevalent genotypes and frequent co-infections in some countries. The first row of Mexico corresponds to our results. HPV 16, 51, and 52 genotypes were commonly reported

Country/Population	HPV genotypes with high prevalence	Frequent co-infections	Ref.
Mexico / Mexico City	*51, 52, 16, and 33* ^a^	*51–52, 16–52, 16–51, and 16-33* ^a^	-
	16, 18, and 68	16–68	[39]
Brazil / Goiânia	16, 51, 31, 52, and 18	16–18	[20]
Kingdom of Bahrain	52, 16, 31, and 51	16–52, 16–31, 16–45, 16–56, and 18–52	[19]
Italy	16, 31, 51, 52, and 6	16–18, 51–52, 16–51-52, and 31–35-56	[38]

^a^Our results

## Conclusions

Based on this data, we concluded that besides HPV 16, the genotypes 33, 51 and 52 are public health concerns and could contribute to cervical carcinogenesis within the Mexican population due to their high frequency. Moreover, the preferential associations among different HPV types (mainly HR-HPV), most likely represent a synergistic interaction in cervical carcinogenesis. These findings call for focusing our research efforts on the clinical implications of the interaction among the different HPV genotypes in co-infections, and for developing new preventive and therapeutic strategies according to the pattern of prevalence in Mexico or other countries.

## Additional files

Additional file 1: HPV genotypes data. The file includes the hybrid capture and genotyping results for each sample and age ranges. The information of gender and age were removed to maintain participant confidentiality. (XLSX 44 kb)

Additional file 2: Graph of interactions among HPV genotypes. The graph corresponds to the data shown in Table 2. Each bar represent the percentage of infected patients (Z axis) with a particular genotype (*X axis, correspond to first column of Table 2) in co-infection with other genotypes analyzed (Y axis). The graphic highlights the strong association between HPV 51 and 52. (TIFF 270 kb)

## Abbreviations

HPV: human papillomavirus
HR-HPV: high-risk human papillomavirus
IARC: The International Agency for Research on Cancer
LR-HPV: low-risk human papillomavirus
PCR: polymerase chain reaction
