# Supplementary figures and images for: High prevalence of co-infection between human papillomavirus (HPV) 51 and 52 in Mexican population
Source: BMC Cancer. 2017 Aug 8;17:531. doi: 10.1186/s12885-017-3519-7 (PMC5549346; doi:10.1186/s12885-017-3519-7)

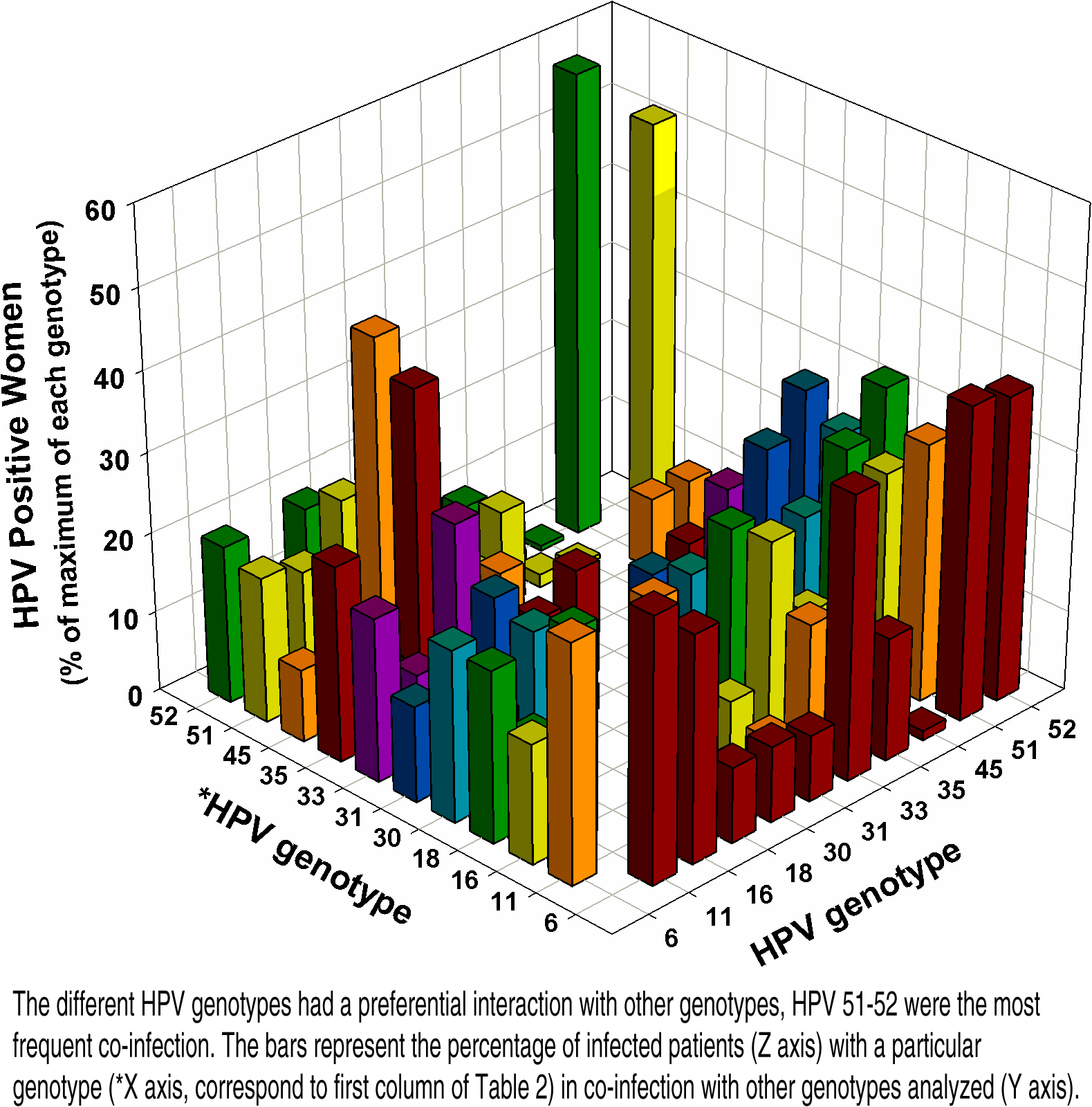

Supplement: Supplementary file 2 — Graph of interactions among HPV genotypes. The graph corresponds to the data shown in Table 2. Each bar represent the percentage of infected patients (Z axis) with a particular genotype (*X axis, correspond to first column of Table 2) in co-infection with other genotypes analyzed (Y axis). The graphic highlights the strong association between HPV 51 and 52. (TIFF 270 kb) [file 12885_2017_3519_MOESM2_ESM.tif]
